# A Cost-Effective Distributed Acoustic Sensor for Engineering Geology

**DOI:** 10.3390/s22239482

**Published:** 2022-12-04

**Authors:** Boris G. Gorshkov, Alexey E. Alekseev, Denis E. Simikin, Mikhail A. Taranov, Konstantin M. Zhukov, Vladimir T. Potapov

**Affiliations:** 1 Prokhorov General Physics Institute of the Russian Academy of Sciences, Vavilov Street 38, 119991 Moscow, Russia; 2 Petrofiber, LLC, Klinsky Proezd, 7, 301664 Novomoskovsk, Russia; 3 Kotelnikov Institute of Radio-Engineering and Electronics, Fryazino Branch, Russian Academy of Sciences, Vvedenskogo Square 1, 141190 Fryazino, Russia

**Keywords:** optical reflectometry, fiber optic sensors, distributed acoustic sensing (DAS)

## Abstract

A simple and cost-effective architecture of a distributed acoustic sensor (DAS) or a phase-OTDR for engineering geology is proposed. The architecture is based on the dual-pulse acquisition principle, where the dual probing pulse is formed via an unbalanced Michelson interferometer (MI). The necessary phase shifts between the sub-pulses of the dual-pulse are introduced using a 3 × 3 coupler built into the MI. Laser pulses are generated by direct modulation of the injection current, which obtains optical pulses with a duration of 7 ns. The use of an unbalanced MI for the formation of a dual-pulse reduces the requirements for the coherence of the laser source, as the introduced delay between sub-pulses is compensated in the fiber under test (FUT). Therefore, a laser with a relatively broad spectral linewidth of about 1 GHz can be used. To overcome the fading problem, as well as to ensure the linearity of the DAS response, the averaging of over 16 optical frequencies is used. The performance of the DAS was tested by recording a strong vibration impact on a horizontally buried cable and by the recording of seismic waves in a borehole in the seabed.

## 1. Introduction

The use of a phase-OTDR or distributed acoustic sensor (DAS) in engineering geology and seismic surveys has been the subject of increased scientific and commercial interest. To date, a significant number of papers have been published describing numerous applications of DAS, including vertical seismic profiling (VSP) [1,2], seismic activity monitoring [3,4,5], subwater seismic activity detection [6,7,8] and surface seismics [9,10]. The advantage of DAS in seismology is the possibility of using standard single-mode or multimode telecommunication fibers in an existing fiber-optic infrastructure, which obtains a huge amount of geological data at relatively low cost. One of the tasks in the current research is to further reduce the complexity and cost of the DAS interrogator, while maintaining high sensor characteristics, such as spatial resolution, low noise level and high fidelity [11].

Most modern DAS systems measure the strain or strain rate of a sensitive fiber that is exposed to external disturbances, with some predefined spatial resolution and strain resolution. These values can be recalculated to the particle velocity of the medium, which is measured by conventional geophones. A more detailed analysis shows that phase-OTDRs measure the phase difference between the fields formed by two separate scattering fiber segments formed by optical pulses in the fiber medium [11,12,13]. The distance between the scattering segments is referred to as the gauge length [14], this parameter defines the spatial resolution of DAS.

The requirements for different DAS applications can significantly differ. VSP measurements require a high spatial resolution (1 m or less) and a wide seismic frequency range; at the same time, the overall cable length is usually not too long (less than 10 km). Engineering geology requires an even shorter cable length—about 1 km. Geological applications, detection of teleseisms and local seismicity require high strain sensitivity and long-term stability, but the acquisition frequency range can be narrowed to tens of Hertz and the optimal spatial resolution can range from 10 to 100 m [15]. At the same time, the total length of the optical cable can be 40 km or more [6].

Recently, a DAS has been proposed that is specialized for use in seismology [8], with active laser frequency stabilization, ensuring long-term stability of the optical frequency and low noise at sub-Hertz frequencies; the strain amplitude noise level was about 0.6 $n\varepsilon /\sqrt{Hz}$ at 0.01 Hz, where nε stands for nanostrain ($10^{-9}$). High performance of the proposed DAS was demonstrated when detecting a remote earthquake using a cable laid on the bottom of the Black Sea. In this paper, a new DAS architecture is considered suitable for VSP, surface seismic applications and engineering geology, having a high spatial resolution of 1 m, high response linearity and an acceptable noise level.

## 2. Dual-Pulse and Single-Pulse DAS Architectures

The basic DAS architectures that can be used for both VSP and other geological applications are described in [14,16]. One of the approaches to creating DAS involves the use of an optical hybrid, usually in the form of a 3 × 3 optical coupler placed in an unbalanced Mach-Zehnder interferometer (MZI) at the receiving side to perform the interferometric demodulation of the backscattered signal. This method was first proposed in [17], and later developed in [18,19]. The feature of this approach is that only one optical pulse is formed and fed into the fiber under test (FUT). This ensures a low level of noise caused by phase fluctuations of the laser source and improves DAS sensitivity [8]. However, this advantage is partially reduced, since the use of a 3 × 3 coupler at the receiving side involves the use of three photodetectors, which ultimately leads to an increase in noise in the demodulated signal. In addition, the MZI is highly susceptible to external thermal and mechanical disturbances, and it is mandatory to provide its thermal and vibration isolation.

Despite some disadvantages, this scheme is often used due to its relative simplicity and reliability. An optical hybrid in the form of a 3 × 3 coupler is a passive, robust and relatively cheap optical component that makes it quite easy to perform quadrature demodulation of backscattered signal.

Another approach to creating DAS, first proposed in [20], involves the use of a dual-pulse probe signal with frequency diversity [20,21] or phase diversity [22] for signal demodulation. The difference in frequencies or phases between the sub-pulses of the dual-pulse can be achieved using an optical phase modulator (PM) placed after the intensity modulator, which is usually an acousto-optic modulator (AOM) [22].

Recently, a new DAS scheme has been proposed, where the phase modulation of the dual-pulse is performed directly by the AOM, which excludes the use of a special phase modulator. The principle of operation was mentioned in [23], and described in detail in [24,25]. In the scheme, the AOM acts not only as the intensity modulator, but also as the phase modulator that provides a phase shift of the optical fields of the sub-pulses in the dual-pulse; the subsequent quadrature demodulation procedure extracts the optical phase change caused by the external fiber disturbance. The value of the phase shift of the optical field in the AOM is controlled by the phase shift of the ultrasonic waves propagating through the crystal [26]. It is essential that the dual-pulse in these schemes is formed by the AOM from continuous wave (CW) light; as a result, the sub-pulses are separated in time and have different phase characteristics owing to inevitable phase fluctuations of the laser source. The resulting interference signal between the backscattered fields produced by the dual-pulse parts contains an excessive noise due to phase mismatch between the sub-pulses of the dual-pulse [27].

Thus, this scheme with an intensity modulator (AOM) dual-pulse formation is inherently noisier than the single-pulse scheme with MZI and 3 × 3 coupler, since the optical path difference between the two parts of the dual-pulse in not compensated. However, only one photodetector is required to detect the backscattered light, which partially compensates for the excessive noise level.

Another advantage of the dual-pulse scheme is that the total energy input into the fiber is twice the energy input in the case of a single-pulse scheme. This obtains a higher average intensity of the backscattered signal before the onset of the limitations associated with nonlinear effects, such as modulation instability. Taking into account the pros and cons of the described schemes, a low noise DAS suitable for engineering geology was proposed, where a dual-pulse is formed from a single-pulse with unbalanced MZI in the beginning of the fiber line [8]. In the proposed scheme, a phase modulator is still required to perform the phase diversity between the sub-pulses of the dual-pulse. In this paper, a dual-pulse DAS without an active phase modulator has been proposed. The phase diversity is achieved by a 3 × 3 fiber coupler, which is placed before the FUT.

## 3. Fading and Nonlinearity of DAS

In all the described DAS schemes, an optical pulse is scattered by inhomogeneities of the fiber refractive index at each moment of time; as a result, multipath interference of backscattered fields with random phases and amplitudes is observed at the receiver. The stochastic nature of the backscattered signal detected by the receiver leads to disadvantages common to all DAS architectures with probing pulses having a narrow spectral linewidth.

Firstly, when random fields are added, it is possible that the amplitude of the total backscattered field is small [14], this situation is typical when adding a large number of coherent fields with random phases and amplitudes, and is called fading. Consequently, the amplitude of the total noise component becomes comparable to the amplitude of the variable part of the backscattered signal; as a result, the detected phase of this total backscattered field is seriously affected by the random phase of the noise component. In this case, the phase change caused by external disturbances cannot be restored without errors.

To eliminate fading, several approaches have been proposed, which are based on receiving the DAS response using various diversity methods, for example, when the responses are obtained at different frequencies of probing pulses [28,29] or with spatial coordinate separation in a multimode optical fiber [30,31].

An effective method for fading reduction is the use of chirped probing pulses [32,33,34,35]. The solution has been claimed to be immune to fading and has a high strain sensitivity up to 3.6 $p\varepsilon /\sqrt{Hz}$ in a frequency range of 200 Hz–5 kHz. The applicability of chirped-pulsed DAS was demonstrated for monitoring teleseismic activity, with the fiber laid on the ocean bottom [6] and earthquake monitoring from a metropolitan area [3].

Another disadvantage of DAS with coherent probing pulses is the inherent nonlinearity of its response to an external disturbance. The question of the linearity of the DAS response was first considered in [16,36], and further contributions to the development of this concept were made in [11,12,13]. The nonlinearity of the DAS response is its intrinsic property, which is based on the fact that the phase increment of the full backscattered field of the scattering fiber segment nonlinearly depends on its strain. This is because the phases of the backscattered fields that make up the total backscattered field change at different rates, depending on the position of the corresponding scattering center in the fiber segment exposed to external strain. However, the phase change of the field backscattered by each individual scattering center within the scattering segment has a linear dependence on the strain of this segment; this can be understood by considering the sum of random phasors on the complex plane [13]. In addition, due to the fact that the scattering amplitudes of the scattering centers within different scattering segments are random, the same strain applied to different fiber segments leads to different and random phase changes of the total backscattered fields, which, moreover, nonlinearly depend on the strain. In short, the same strain applied to different segments of the fiber leads to different nonlinear responses.

In general, the DAS response can be represented as the sum of linear and nonlinear components with respect to strain of the fiber [11,12,13]. The linear part of the DAS response is of interest, as it is attributed to the optical phase change within the gauge length (the distance between the scattering segments). There are two main approaches to reducing the nonlinearity of the DAS response [36]. The first approach is based on increasing the ratio of the gauge length to the length of individual scattering segments. This can be achieved either by reducing the duration of the optical pulse and, accordingly, the spatial extent of the scattering segment, or by increasing the gauge length. In the first case, the energy of the probing pulse decreases, and, consequently, the optical signal-to-noise ratio of the backscattered signal also decreases. In the second case, the spatial resolution of DAS suffers, so high seismic frequencies are accordingly suppressed.

The second approach is based on averaging the DAS response over multiple optical interrogation frequencies [28,29,37]. The idea of linearization of the DAS response is that, for different optical frequencies of the probing light, the nonlinear components of the responses are different, while the linear components remain unchanged. As shown in [13], the DAS response averaged over independent response realizations is linear with respect to the external action. Thus, averaging over several DAS responses obtained at different optical frequencies can reduce the random nonlinear contribution and highlight the linear contribution, provided that the single responses are statistically uncorrelated. The latter condition is met if the difference in the optical frequencies of the probing pulses exceeds the inverse of the pulse duration [38]. The linearization of the DAS response when averaging over 20 optical frequencies with weighting coefficients is discussed in [37]; a high degree of linearity was achieved, at which the coefficient of nonlinear distortion (Total Harmonic Distortion, THD) was −40 dB, and fading was also effectively eliminated.

The described DAS response nonlinearity is related to the finite size of the scattering segment. Therefore, when using a fiber with point reflectors in the form of ultra-weak fiber Bragg gratings [39] or enhanced backscattering fiber [40], these nonlinear effects are minimized.

The DAS architecture described in the next section implies either impact averaging (accumulation over multiple impacts), or averaging responses at different optical interrogation frequencies. This makes it possible to effectively reduce the fading and increase the linearity of the DAS response relative to external actions.

## 4. Proposed DAS Architecture

**Figure 1:**
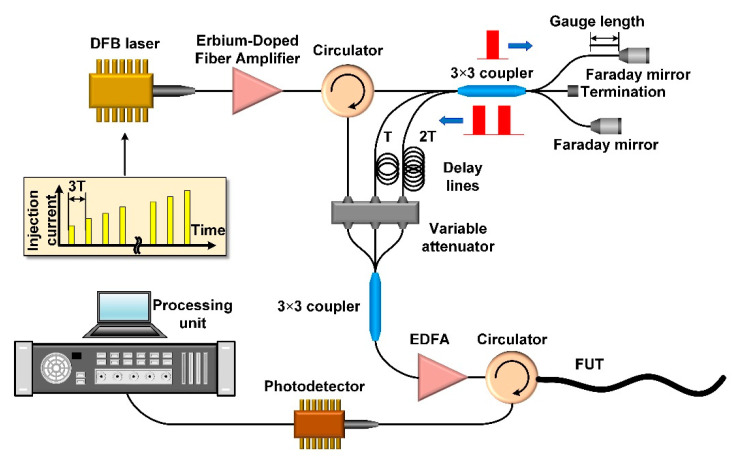
The principal scheme of the proposed DAS. Three pairs of dual-pulses with a phase shift are formed in an unbalanced MI, then, they are appropriately delayed and sequentially enter the FUT. The backscattered light is demodulated in the processing unit.

The principal scheme of the proposed DAS is shown in Figure 1. A distributed feedback laser (DFB) diode Fujitsu FLD5F8HF LK with a wide spectral linewidth of about 1 GHz, operating in the regime of direct modulation of the injection current, generates optical pulses with a duration of 7 ns (FWHM) and at different frequencies.

The pulses are amplified and injected into the unbalanced MI through an optical circulator. The MI consists of a symmetrical 3 × 3 fiber-optic coupler with two arms of different lengths; the third port of the coupler is terminated. An unbalanced MI generates three dual-pulses from a single input pulse with a delay between the sub-pulses determined by the difference in the MI arm lengths, which in turn, determines the DAS gauge length. The use of Faraday rotation mirrors ensures the matching of polarization states of the sub-pulses.

The property of the 3 × 3 coupler is that the fields injected into it from different input ports are summed up in output ports with different relative phases [17,18,19]. If the 3 × 3 coupler is symmetric, then the relative phase delay between the optical fields at the output is equal to 2π/3. As a result, at the output ports of the 3 × 3 coupler, dual-pulses with relative phase differences equal to $\begin{array}{ccc} 0, & 2\pi /3, & -2\pi /3 \end{array}$ between sub-pulses are formed. Furthermore, the dual-pulse from the first port of the 3 × 3 coupler enters the second 3 × 3 coupler, which in this case acts as an optical combiner, then, it is amplified and injected into the FUT. The dual-pulse from the second port of the 3 × 3 coupler additionally passes through the delay line, the length of which must exceed twice the length of the FUT (the time delay is denoted as T in Figure 1), after which it also enters the combiner, is amplified, and enters the FUT. The dual-pulse from the third port of the 3 × 3 coupler passes through the delay line, the length of which must exceed four times the length of the FUT (the time delay is denoted as 2T in Figure 1), after that, it also enters the combiner and, after amplification by the Erbium Doped fiber Amplifier (EDFA), enters the FUT. Optionally, the power levels of the three dual-pulse groups can be aligned using the attenuation unit.

Thus, by analogy with our previously proposed DAS configuration [22], the setup described above generates three dual-pulse groups with a relative phase delay between sub-pulses. The backscattered light is detected by the avalanche photodiode with the band consistent with the spectral width of the OTDR trace and demodulated using the algorithm based on the Kalman filter [41].

The DAS architecture, where the dual-pulse is formed by the unbalanced interferometer in the beginning of the FUT, as well as the architecture where the unbalanced interferometer is placed at the end of FUT (i.e., is installed ahead of the photodetector), has an advantage over the architecture where a dual-pulse is formed by an intensity modulator or by the source itself. In all these cases, an interference signal of light backscattered by two separate fiber segments is obtained. However, in the interferometer-based DAS schemes, the sub-pulses of the dual-pulse are coherent with each other, since they are generated by the MI from a single input pulse. The time delay between sub-pulses introduced by MI is compensated in the FUT; the absence of this delay makes the DAS response less sensitive to the frequency drift of the probing light and leads to the reduction of noise caused by the laser light phase fluctuations [8,27]. On the contrary, in the dual-pulse DAS schemes without an interferometer, where the pulses are formed by an intensity modulator from CW laser light, the sub-pulses of the dual-pulse are less coherent as the temporal delay between the sub-pulses always exists, which leads to a dependence of the DAS response on frequency stability and a stronger dependence on the coherence properties of the source.

Another advantage of using the dual-pulse DAS architecture already mentioned above is that, in this case, twice as much optical energy can be injected into the optical fiber below the threshold of nonlinear effects, particularly for modulation instability. The threshold of this effect is inversely proportional to the FUT length, thus, for long FUTs (more than 6 km), it has a value of about 200 mW for fibers with anomalous dispersion [42], while for shorter fiber lines it increases to about a watt [33]. In the described DAS with the FUT length equal to 1 km, it has the experimentally confirmed value of 1250 mW. In addition, the proposed DAS requires only one photodetector, which reduces the complexity of the architecture and increases its reliability.

Direct modulation of the laser injection current enables the generation of short pulses with a duration of about several nanoseconds. This leads to an increase of the laser spectral linewidth and an increase in the intensity noise in the backscattered signal. The tuning of the injection current amplitude allows for changing the frequency of laser light in such a way that the introduced frequency offset is enough to decorrelate the sequential OTDR traces [38]. In the experiments, 16 optical interrogation frequencies were used for sequential triples of dual-pulses. After receiving and demodulating the backscattered light at each laser frequency, a set of independent DAS responses is obtained. The subsequent averaging of the responses minimizes fading and nonlinearities [13,37]. To improve the signal quality, various averaging procedures can be used. The responses can be averaged without any weighting factors [13] but, in practice, averaging with weighting coefficients is more useful, since in this case the influence of noisy spatial channels can be reduced, while the linearity of the result is acceptable. One of the approaches is the averaging of DAS responses with weighting factors proportional to the amplitudes of the backscattered fields [29,37].

Another advantage of the proposed scheme is that there is no need to use an additional intensity modulator, such as an acousto-optic modulator (AOM), an electo-optic modulator (EOM), or a semiconductor optical amplifier (SOA), to generate short dual-pulses, which significantly reduces the cost of the DAS. Furthermore, the required phase shifts between the parts of the dual-pulse are obtained by using a simple passive 3 × 3 coupler. This makes the DAS architecture simple and cost-effective.

The obvious disadvantage of the proposed scheme is the requirements for temperature and vibration isolation of the interferometer used, due to its high sensitivity to vibrations and temperature change. Any disturbances to the interferometer distort the DAS response. This drawback, however, is easily eliminated by using a special sealed enclosure [8]. As can be seen in Figure 1, for the sequential injection of three groups of dual-pulses in the FUT, it is necessary to additionally organize two delay lines that determine the maximum possible length of the FUT; these delay lines, however, do not require temperature or vibration stabilization.

To obtain an averaged DAS readout, it is necessary to use probing pulses at several optical frequencies, this proportionally reduces the maximum frequency of a detectable acoustic signal. Probing pulses at various optical frequencies were obtained by changing the injection current of a laser diode. In the proposed DAS, a simple digital data acquisition card (DAQ) is used with a sampling rate of 100 Ms/s and 16 different optical frequencies; the sampling frequency for a 1 km long FUT is up to 2 kHz, which is often enough for engineering geology.

**Figure 2:**
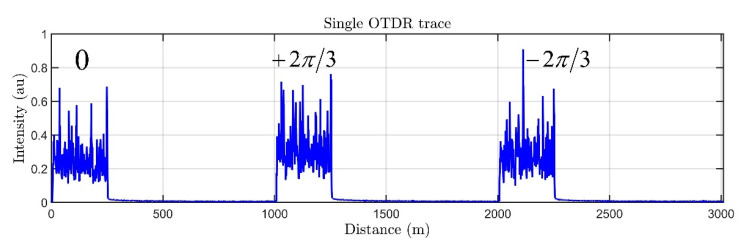
A typical DAS OTDR trace. Three groups corresponding to different phase shifts are shown.

A typical OTDR trace of the DAS is shown in Figure 2, it consists of three consecutive OTDR traces corresponding to three dual-pulses with different phase shifts between sub-pulses.

## 5. Experiments

**Figure 3:**
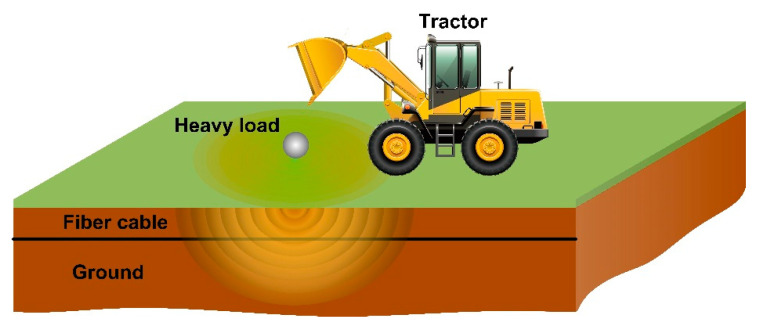
Registration of waves excited when a heavy load falls to the ground.

The applicability of the proposed DAS has been demonstrated in two experiments. The first experiment was the detection of waves produced by the hit of a heavy load on the ground, as shown in Figure 3.

A tractor lifted a load weighing 200 kg to a height of two meters and dropped it to the ground at a distance of about 10 m from the buried cable. The sensing fiber cable was buried in the ground to a depth of 50 cm in an industrial environment. The two fibers in the cable were sequentially spliced at the end of the cable.

**Figure 4:**
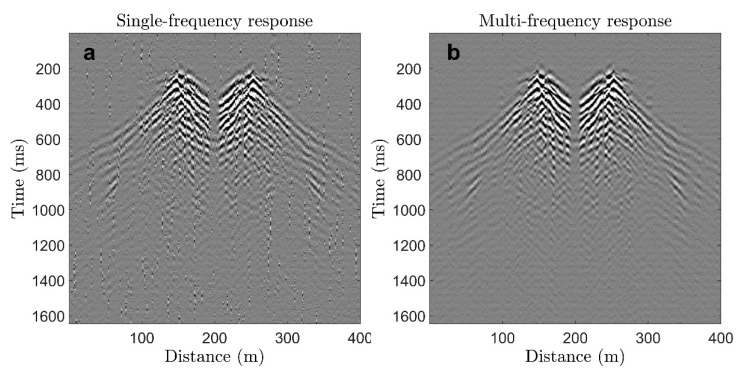
The DAS response in space-time domain at a single interrogation frequency (**a**), and averaged over 16 interrogation frequencies (**b**). The mirror symmetry is due to the fact that the two fibers in the cable were spliced at the end of the cable. Grayscale shows the amplitude of seismic waves in arbitrary units. The record starts approximately 200 ms prior to the load hitting the ground.

Propagating waves were recorded using the described DAS interrogator with a duration of dual-pulse parts equal to 7 ns and a gauge length equal to 1 m. The number of interrogation frequencies were 16 and the sampling rate was 2 kHz. Figure 4 shows the results of the experiment; i.e., the propagation of seismic waves in the ground. The DAS response at a single frequency is shown in Figure 4a; the DAS response averaged over 16 frequencies is shown in Figure 4b.

**Figure 5:**
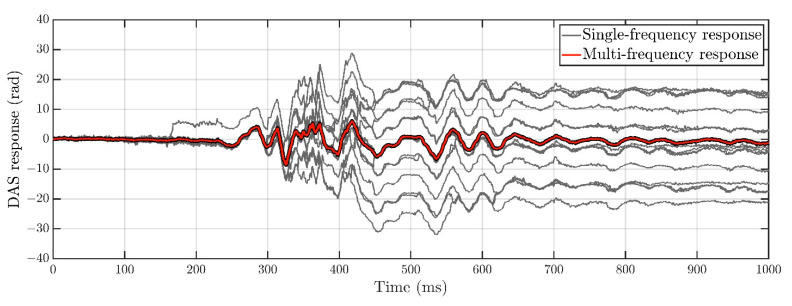
The DAS response in one spatial channel at 16 interrogation frequencies with visible phase jumps (gray lines) and the average DAS response (red line). Phase jumps are eliminated after averaging.

The DAS response has a satisfactory appearance even at one interrogation frequency, but the image is noisy with visible phase jumps, which occur due to fading. Averaging noticeably improves the signal-to-noise ratio and reduces the number of phase jumps. This improvement can be seen in Figure 5, which shows several DAS responses in one spatial channel received at 16 different interrogation frequencies with visible phase jumps [13], as well as the average response in which phase jumps are eliminated.

**Figure 6:**
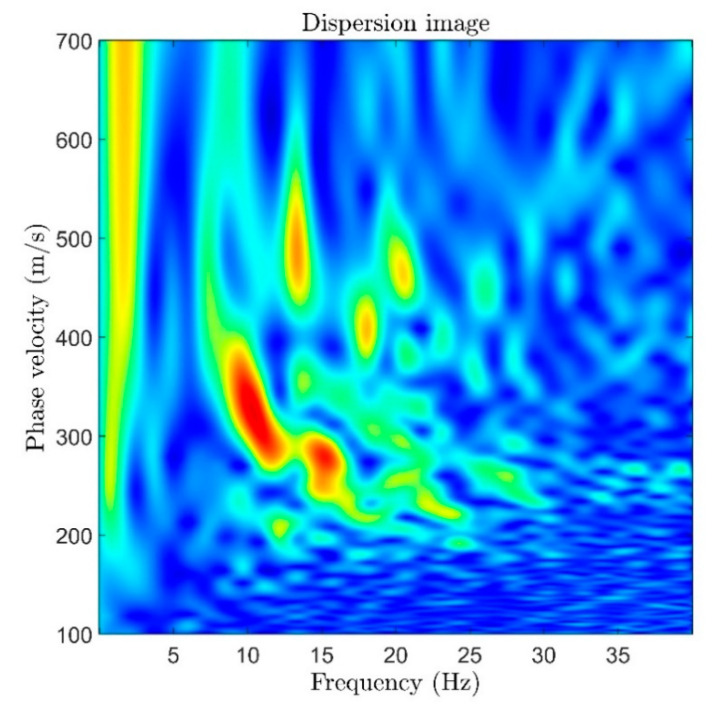
Phase velocity dispersion image of the seismic field record shown in Figure 4. The dark blue color corresponds to the lowest magnitude, the red color corresponds to the highest magnitude.

The obtained seismic field record can be used to construct a velocity profile of the near-surface layers of the medium. e.g., using multichannel analysis of surface waves (MASW) [43]. The phase velocity dispersion image obtained from data in Figure 4b is shown in Figure 6.

The dispersion image in Figure 6 shows that surface waves with a frequency of about 10 Hz and a phase velocity of about 320 m/s dominate in the resulting record of the seismic field. Further inversion process obtains the velocity profile of the medium [43].

**Figure 7:**
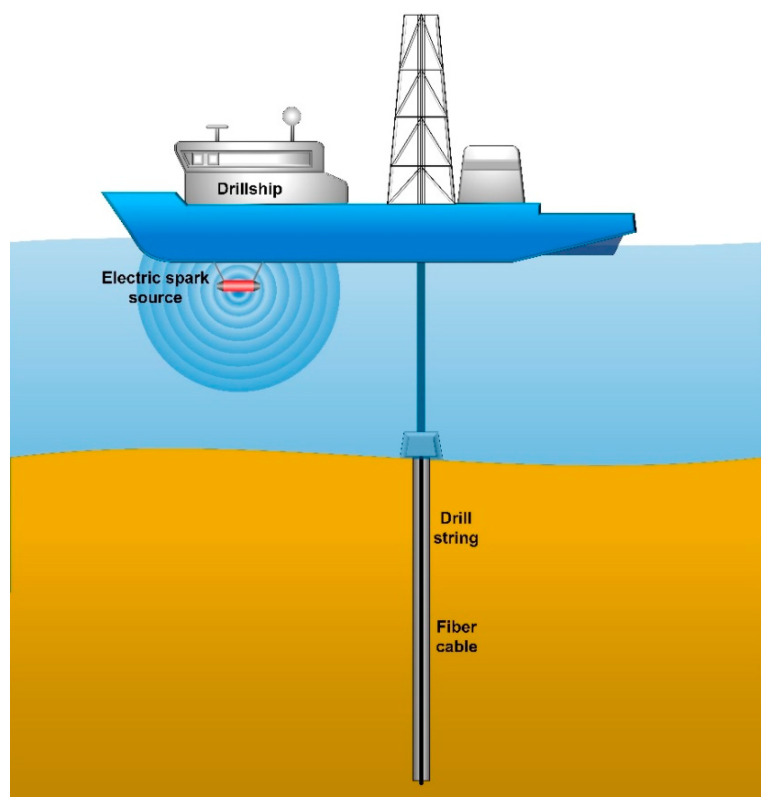
Zero-offset vertical seismic profiling (ZVSP) obtained in the Laptev Sea.

The second experiment with the proposed DAS was a zero-offset vertical seismic profiling (ZVSP) obtained in the Laptev Sea, as shown in Figure 7.

The detailed description of the experiment is given in [44]. A well with a depth of 130 m was drilled from a drillship in the seabed. The free-hanging FUT of the DAS was deployed into the drill string. An electric spark source located at a depth of about 1 m underwater excited hydro waves with a central excitation frequency of 260 Hz and a frequency range from 100 to 400 Hz. The impacts were performed once per second having an output energy of 2250 J. The hydro waves excited seismic waves in the seabed, which were detected by the FUT in the well. The excitation source was operated for 1 h and performed 3600 acoustic impacts. The distance from the source to the wellhead was about 15 m.

In this experiment, an ECL (external cavity laser) diode with a spectral linewidth of about 100 kHz was used. The reason for choosing a laser with a higher degree of coherence in this case is due to the requirement to ensure a lower noise level of the received backscattered signal and detect tiny changes in the backscattered intensity. The total backscattered intensity noise power, in this case, is determined by the ratio of the coherence length to the pulse length [27]. In addition, only one frequency of the probe pulse was used, which increased the sampling rate to 30 kHz. The DAS gauge length was 2 m and the duration of the sub-pulse in the dual-pulse was 7 ns. Instead of averaging over different interrogation frequencies, DAS responses were averaged over different impact realizations. These measures made it possible to register seismic signals with low amplitudes.

**Figure 8:**
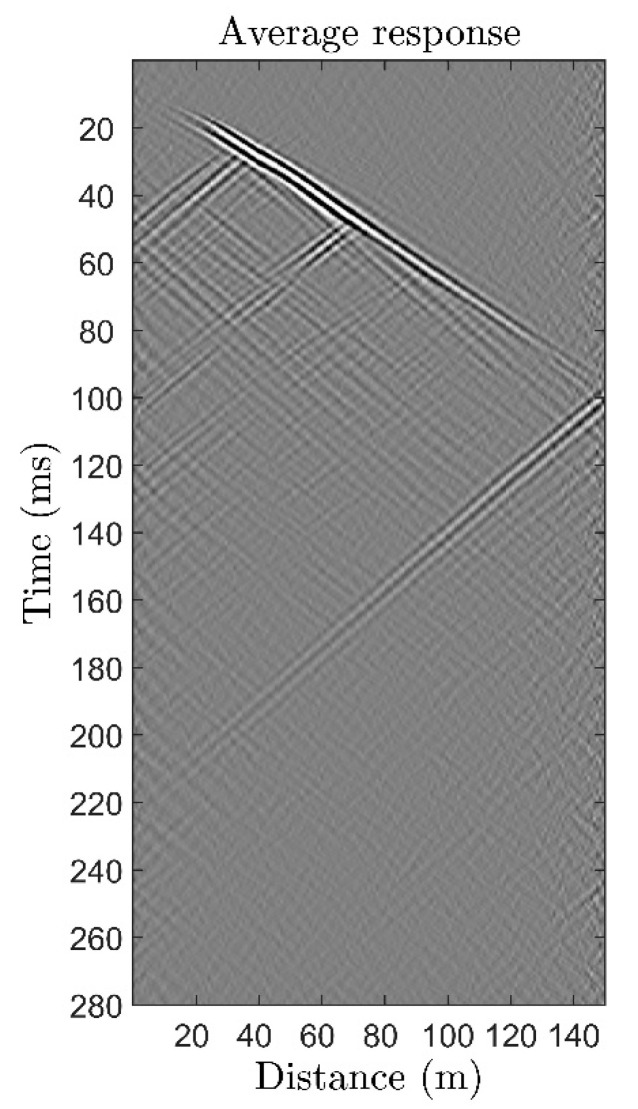
The result of ZVSP using the proposed DAS with averaging over 3600 acoustic impacts. The waveform was f-k-filtered to remove hydroacoustic waves and environmental noise.

Due to the imperfect contact of the cable and the drill string, the single measurement response was not of good quality. The subsequent averaging of 3600 measurements significantly improved the signal-to-noise ratio. The results of averaging over 3600 measurements are shown in Figure 8. The waveform was additionally filtered with an f-k filter to remove hydroacoustic waves, tube waves, and environmental noise. The quality of the data obtained was sufficient for use in further geological research, which indicates great potential for using the proposed DAS in geological marine surveys.

## 6. Conclusions

A new DAS architecture based on an unbalanced MI and a 3 × 3 fiber coupler is proposed. The difference between the proposed scheme and conventional optical hybrid approaches in DAS is that the 3 × 3 coupler is used as part of the unbalanced MI before the FUT in the dual-pulse formation scheme. The 3 × 3 coupler acts as an optical phase shifter, which allows for the use of conventional quadrature demodulation procedures of backscattered light. The sub-pulses of the dual-pulse are coherent with each other as they are produced from a single optical pulse. The delay between the sub-pulses introduced by MI compensates for the delay between fields backscattered from different fiber segments of the FUT.

Two experiments were conducted, demonstrating the operation of the proposed DAS scheme. In the first experiment, a strong impact caused by a heavy load onto the ground was detected. In the DAS setup, a relatively inexpensive laser with a wide spectral linewidth of 1 GHz was used. Direct modulation of the laser injection current made it possible to sequentially obtain probing pulses at 16 different frequencies. Averaging responses over 16 different frequencies further increased the signal-to-noise ratio of the DAS response.

In the second experiment, seismic waves were recorded in a borehole located in the seabed. The acoustic impact was formed by an electric spark source located near the surface of the water. Because the amplitude of seismic waves affecting the cable in the drill string was relatively small, a highly coherent laser was used as a source of probe light. Averaging was performed over 3600 independent shots; as a result, an acceptable signal-to-noise ratio in the averaged response was obtained for the refinement of the medium geological model.

The experiments carried out demonstrate the operability of the proposed DAS scheme and its flexibility for use in completely different types of research, with the possibility of using lasers of various types and different types of averaging. The simplicity and cost-effectiveness of this DAS architecture opens up great opportunities for its use in a wide range of tasks.

## Data Availability

The data presented in this study are available on request from the corresponding author.
